# Genome-wide identification and expression analysis of C_3_HC_4_-type RING finger gene family in *Gossypium hirsutum*

**DOI:** 10.3389/fpls.2026.1789503

**Published:** 2026-05-12

**Authors:** Xue Song, Shuke Yang, Lili Zhang, Chaofeng Hao, Fan Li, Hongwei Sun, Xiuna Guo, Hongxia Zhang, Xingbo Lu, Xiaohui Xu, Xinyao Xia

**Affiliations:** 1 Shandong Key Laboratory for Green Prevention and Control of Agricultural Pests, Institute of Plant Protection, Shandong Academy of Agricultural Sciences, Jinan, China; 2 Key Laboratory for Safety Assessment (Environment) of Agricultural Genetically Modified Organisms, Ministry of Agriculture and Rural Affairs, Jinan, Shandong, China; 3 College of Horticulture (The Engineering Research Institute of Agriculture and Forestry), Ludong University, Yantai, Shandong, China

**Keywords:** abiotic stress, biotic stress, C_3_HC_4_-type RING finger gene family, cotton, tissue-specific expression

## Abstract

The C_3_HC_4_-type RING finger (Ring-HC) gene family encodes zinc finger proteins crucial for plant growth and stress resistance. However, a comprehensive study on C_3_HC_4_-type RING finger genes in cotton has not been conducted to date. We identified 56 C_3_HC_4_-type RING finger genes in *G. hirsutum*, classified into three subfamilies with conserved structures. Evolutionary analysis indicated that this gene family has undergone strong purifying selection. Expression profiling revealed distinct tissue specificity, with significant enrichment in roots and ovules, indicating potential roles in development. Under abiotic stress, *GhRHC* genes showed broad responsiveness, particularly to cold. Notably, members like *GhRHC1* and *GhRHC34* exhibited antagonistic expression patterns: upregulation during pathogen/pest attacks but downregulation during beneficial rhizobacterial colonization. qRT-PCR analysis further confirmed the cold-induced expression of *GhRHC1* and *GhRHC34*. Subcellular localization results preliminarily indicated that GhRHC1 and GhRHC34 proteins are localized to the nucleus and the cytoplasm. Our findings suggest that the *GhRHC* family plays multifaceted roles in cotton development and stress adaptation. The differential regulation of specific members suggests a possible association between differential stress-responsive regulation and the balance between defense-related responses and beneficial rhizobacterial interactions.

## 1 Introduction

Zinc finger transcription factors represent one of the most extensive families of transcription factors (TFs) in plants, playing a pivotal role in diverse metabolic processes, including DNA recognition, RNA modification, cell differentiation, apoptosis, and protein-protein interactions (Dutta et al., 2022). Accumulating evidence has demonstrated that zinc finger proteins play crucial roles in plant growth, development, and responses to environmental stress (Han et al., 2020). Zinc finger proteins are categorized based on the number and position of cysteine (C) and histidine (H) residues coordinating with Zn^2+^ ions within the zinc finger domain, resulting in nine primary types: C_2_H_2_, C_2_HC, C_2_HC_5_, C_3_H, C_3_HC_4_, C_4_, C_4_HC_3_, C_6_, and C_8_ (Han et al., 2020). Among these, C_3_HC_4_ zinc finger proteins are distinguished by the presence of RING (Really Interesting New Gene) domains (Yuan et al., 2013). This designation arises from their conserved arrangement of cysteine and histidine residues (Cys-X2-Cys-X(9-39)-Cys-X(1-3)-His-X(2-3)-Cys-X2-Cys-X(4-48)-Cys-X2-Cys, where X denotes any amino acid). The RING domain forms a stable ring structure through zinc ion chelation, facilitating interactions with various molecules. These proteins are integral components of the ubiquitin-proteasome system (UPS), primarily functioning as E3 ubiquitin ligases to mediate substrate ubiquitination—a process critical for regulating protein degradation, signal transduction, and cellular homeostasis (Sun et al., 2019).

As an E3 ubiquitin ligase, RING-HC proteins play critical roles in plant growth and development (Kou et al., 2024). For instance, RING-HC proteins inhibit the photomorphogenesis of Arabidopsis thaliana under light conditions and modulates root elongation in response to jasmonic acid. Additionally, it influences the fruit development of tobacco (Chen et al., 2024) and affects bud growth, silique formation, and seed maturation in Arabidopsis thaliana (Qi et al., 2017). Furthermore, the RING-HC protein is implicated in plant responses to both biotic and abiotic stresses (Gao et al., 2012). For example, studies in Arabidopsis thaliana and chili peppers have shown that the RING-HC protein is actively involved in abscisic acid synthesis, thereby negatively regulating the drought response (Zhao et al., 2021). *RZFP1* in Chinese cabbage is implicated in responses to salt, dehydration, and cold stress. Under biotic stress conditions, *ATL9* in Arabidopsis thaliana and *RHC1* in rice facilitate immune responses through the ubiquitination and degradation of pathogenic inhibitory proteins (Chen et al., 2024). These studies suggest that C_3_HC_4_-type zinc finger proteins play a crucial role in plant growth and development, as well as in conferring resistance to abiotic stress (Chen et al., 2025). Cloning and functional characterization of these proteins across a wider range of plant species will enhance our understanding of the mechanisms underlying plant abiotic stress tolerance (Han et al., 2021).

The global annual production of cotton is approximately 25 million tons (Khan et al., 2020). As a major textile fiber and oilseed crop, cotton plays a crucial role in supporting the livelihoods of over 95 million individuals in developing countries (Zafar et al., 2020). While the introduction of the *Bacillus thuringiensis* (Bt) gene has significantly mitigated the impact of lepidopteran pests on cotton, the crop continues to encounter various other adverse environmental conditions during its growth and development, such as drought and high salinity (Shahrajabian et al., 2020). These abiotic stress factors present substantial challenges to the growth, development, and yield optimization of cotton. Consequently, it remains essential to investigate the molecular mechanisms underlying cotton’s response to these adverse stressors and to enhance its resilience (Patil et al., 2024). *Gossypium hirsutum* was selected for genome-wide analysis because it is the most extensively cultivated cotton species worldwide and possesses well-annotated genome and abundant publicly available transcriptomic datasets under diverse abiotic and biotic stresses. These advantages enable comprehensive functional inference and enhance the potential application of identified candidate genes in cotton improvement programs.

In this study, we performed a comprehensive genome-wide analysis of the C_3_HC_4_-type RING finger gene family in cotton, providing an extensive overview that includes phylogenetic relationships, motif compositions, and potential cis-elements. We also examined the differential expression profiles of this gene family across various tissues. Our findings provide a foundation for future research into the roles of these candidate genes in cotton growth, development, and stress responses, as well as for genetic engineering and functional genomics studies.

## 2 Materials and methods

### 2.1 Identification of C_3_HC_4_-type RING finger genes in cotton

The comprehensive genomic dataset for *Gossypium hirsutum* (version 2.1) was obtained from the National Center for Biotechnology Information (NCBI) database (https://www.ncbi.nlm.nih.gov/). To identify potential C_3_HC_4_-type RING finger members within the cotton genome, the BLAST (Henzinger et al., 2003) and HMMER (Finn et al., 2011) software tools were employed, utilizing an E-value threshold of 10^-5^. The definitive RHC members, characterized by the presence of a C_3_HC_4_-type RING finger domain, were subsequently validated through the NCBI Conserved Domain Database (NCBI-CDD).

### 2.2 Conserved motifs identification and phylogenetic analysis

Multiple sequence alignments of confirmed C_3_HC_4_-type RING finger proteins were conducted using ClustalW (Larkin et al., 2007) with default settings. Phylogenetic trees of these protein sequences were subsequently constructed utilizing the MEGA software (version 7.0.21) (Tamura et al., 2007) employing the neighbor-joining (NJ) method with 1000 bootstrap replications. Cotton C_3_HC_4_ members were named based on their phylogenetic relationships with corresponding members from rice and poplar. The phylogenetic trees were visualized using the online tool ITOL (https://itol.embl.de/) (Letunic and Bork, 2024). Conserved motifs within the C_3_HC_4_-type RING finger genes were identified using the MEME program (http://meme-suite.org/tools/meme) (Bailey et al., 2006), applying an E-value threshold of <0.05. Sequences that were excessively short or lacked the necessary domains were excluded from the analysis.

### 2.3 Gene location and duplication analysis

The locational data for C_3_HC_4_-type RING finger members were derived from genome annotation files. Subsequently, Mapchart (Voorrips, 2002) was employed to illustrate the chromosomal positions of these genes. Prior to conducting collinearity analysis, each cotton genome underwent self-comparison using BLAST. The segmental and tandem duplication landscapes were assessed using MCScanX (Wang et al., 2012). In-house Perl scripts were used to filter duplicate segments containing cotton C_3_HC_4_ members. Finally, CIRCOS was utilized to construct the gene location and synteny map (Krzywinski et al., 2009).

### 2.4 Expression analysis based on the transcriptomics data

The expression profiles of C_3_HC_4_-type RING finger genes were re-evaluated utilizing existing cotton datasets from the Plant Public RNA-Seq Database (https://plantrnadb.com/) (Yu et al., 2022). An in-house Perl script was employed to extract the expression data specific to C_3_HC_4_-type RING finger members from the comprehensive dataset. The fragments per kilobase million (FPKM) values of the NRT genes were normalized to dimensionless data, and bubble plots were generated using TBtools (Chen et al., 2023). Differentially expressed genes were identified using the R packages limma (Smyth, 2005) and GEOquery (Davis and Meltzer, 2007). Furthermore, principal coordinates analysis (PCoA) with pairwise Adonis test and PERMANOVA (999 permutations) were conducted on the expression data with the aid of the R packages (version 4.1.1) ggplot2 (Wickham, 2011), vegan (Oksanen, 2015), and ggbiplot (Vu et al., 2011).

### 2.5 Quantitative real-time PCR analysis

The expression of *GhRHC1*, *GhRHC7*, *GhRHC18*, *GhRHC29*, *GhRHC34* and *GhRHC48* under cold stress were confirmed by qRT-PCR. The cotton cultivar ICR 49 (Institute of Cotton Research 49, CNA20050567.X) was employed as the experimental material. Briefly, ICR 49 was planted in greenhouse of Shandong Academy of Agricultural Science. The cotton was transferred to the pre-cooling growth chamber for 24 h (8°C for 12h and 4°C for 12h) when cotton seedlings reached the three-leaf and one-terminal-bud stage. Cotton planted in the environment at 25°C for 24 h served as the control. After a 24-hour cold treatment, cotton leaves were sampled for RNA extraction. TRIzol reagent (Invitrogen, Carlsbad, CA) and the RNA MiniPrep kit (Zymo Research, Irvine, CA) were used to extract the total RNA. Then, the RNA was examined by agarose gel electrophoresis. Up to 1 μg of total RNA was utilized to synthesize cDNA using the HiScript III 1st Strand cDNA Synthesis Kit from Vazyme, Nanjing, China. A 20 μl volume was used for qRT-PCR, consisting of 5 μl of diluted cDNA template, 0.5 μl of each specific primer, 10 μl of RealStar Green Fast Mixture with ROXII (GenStar, Beijing, China), and 4 μl of ddH2O. The PCR cycle settings included an initial step at 95 °C for 10 minutes, followed by 40 cycles of 95 °C for 10 seconds and 60 °C for 30 seconds. *GhSad1* was used as stable reference genes. The primer pairs specific to each gene for qRT-PCR were created using NCBI Primer BLAST and are listed in **Supplementary Table 1**. To guarantee the accuracy of the results, three biological replicates were conducted. Relative expression of the target genes was calculated using the 2^−ΔΔCt^ method (Livak and Schmittgen, 2001). Significant differences in gene expressions between cold treatment and control groups were evaluated by a two-tailed unpaired Wilcoxon rank sum test at a threshold P-value < 0.05.

### 2.6 Subcellular localization of *GhRHC1* and *GhRHC34*

The full-length coding sequences (CDS) of *GhRHC1* and *GhRHC34* genes without termination codons were cloned into the pCAMBIA1302 vector with an enhanced green fluorescent protein (GFP) tag under the 35 S promoter via LR reaction. The recombinant constructs and the empty GFP vectors were introduced into *Agrobacterium tumefaciens* (GV3101-p19) and infiltrated into the leaf epidermal cells of tobacco (*Nicotiana benthamiana*). After 72 h of growth under 28 °C with a 12 h light and 12 h dark cycle, the subcellular localization of *GhRHC1* and *GhRHC34* were observed by the Nikon-C2 confocal microscope (Nikon, Tokyo, Japan) with four independent biological replicates. Primer sequences used for vector construction are listed in **Supplementary Table 2**.

## 3 Results

### 3.1 Identification and phylogenetic analysis of C_3_HC_4_-type RING finger genes in *G. hirsutum*

**Figure 1:**
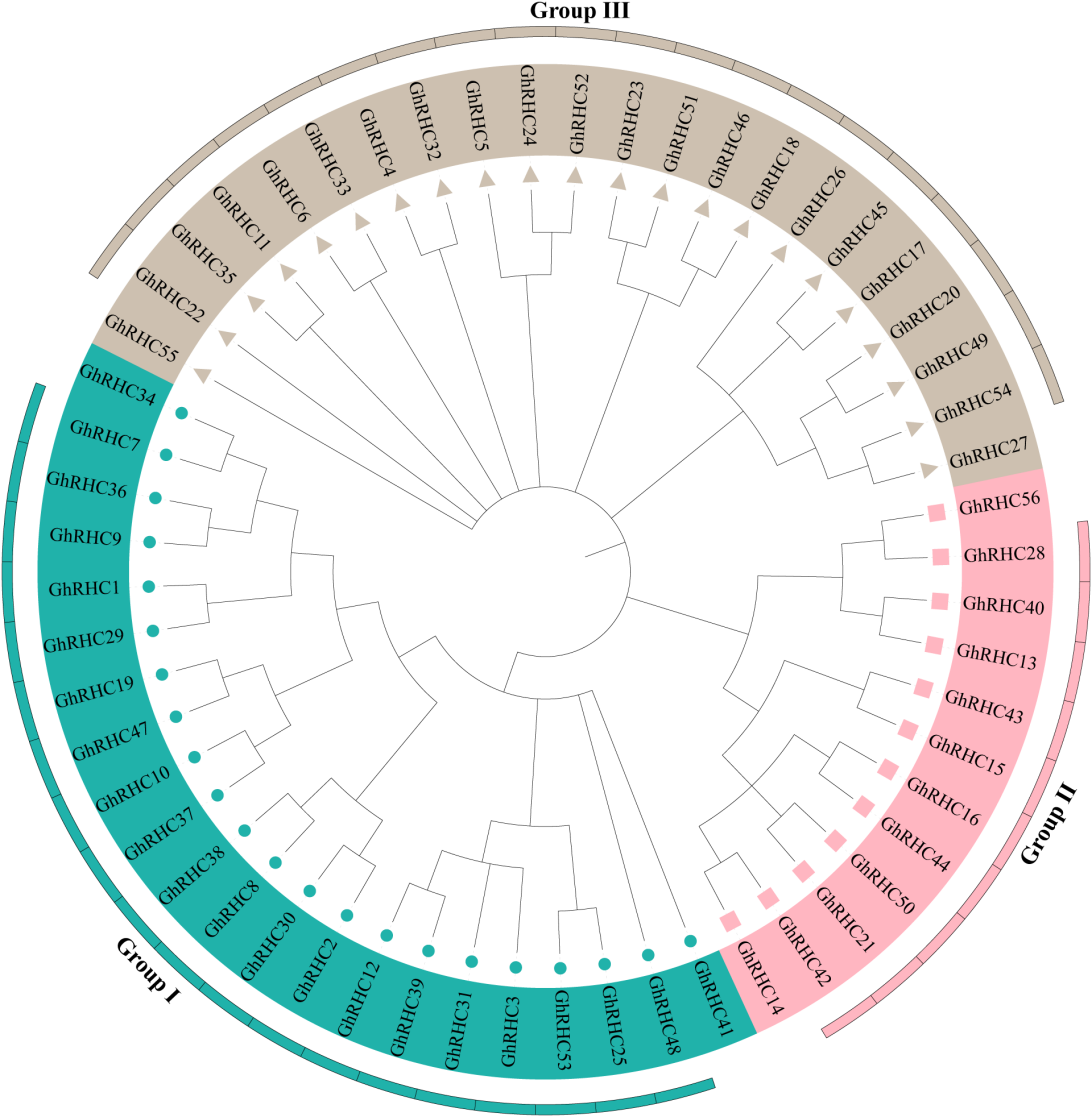
Phylogenetic tree of C_3_HC_4_-type RING finger gene family in cotton. The C_3_HC_4_-type RING finger genes in different groups were marked with different colors. Phylogenetic trees were built using MEGA7 with Neighbor-Joining method and bootstrap of 1000 replications.

C_3_HC_4_-type RING finger proteins from rice and poplar were utilized as reference sequences to conduct a BLASTP search within the cotton genome, employing an E-value threshold of 10-5 (**Supplementary Table 3**). Domain confirmation via NCBI-CDD led to the identification of 56 C_3_HC_4_-type RING finger genes in *G. hirsutum* (**Supplementary Table 4**). The average number of introns per subfamily ranged from one in RHC-I to eight in RHC-III and 14 in RHC-II. Notably, substantial divergence in amino acid sequence length was observed among the three subfamilies. Specifically, the average protein length in the RHC-I subfamily was 247 amino acids, significantly shorter than the average lengths in RHC-II (711 amino acids) and RHC-III (626 amino acids). A similar pattern of length variation was observed in the C_3_HC_4_-type RING finger genes. The average gene length within the RHC-I subfamily is 2174 base pairs (bp), which is considerably shorter than that of the RHC-II (7356 bp) and RHC-III (5678 bp) subfamilies. To investigate the phylogenetic relationships among the C_3_HC_4_-type RING finger genes in cotton, a phylogenetic tree was constructed using the full-length protein sequences of 56 identified C_3_HC_4_-type RING finger genes. As illustrated in **Figure 1**, these genes are categorized into three distinct groups: Group I, Group II, and Group III. Notably, Group I and Group III each comprise 22 members, whereas Group II consists of only 12 members.

### 3.2 Chromosomal distribution of the C_3_HC_4_-type RING finger members in cotton

**Figure 2:**
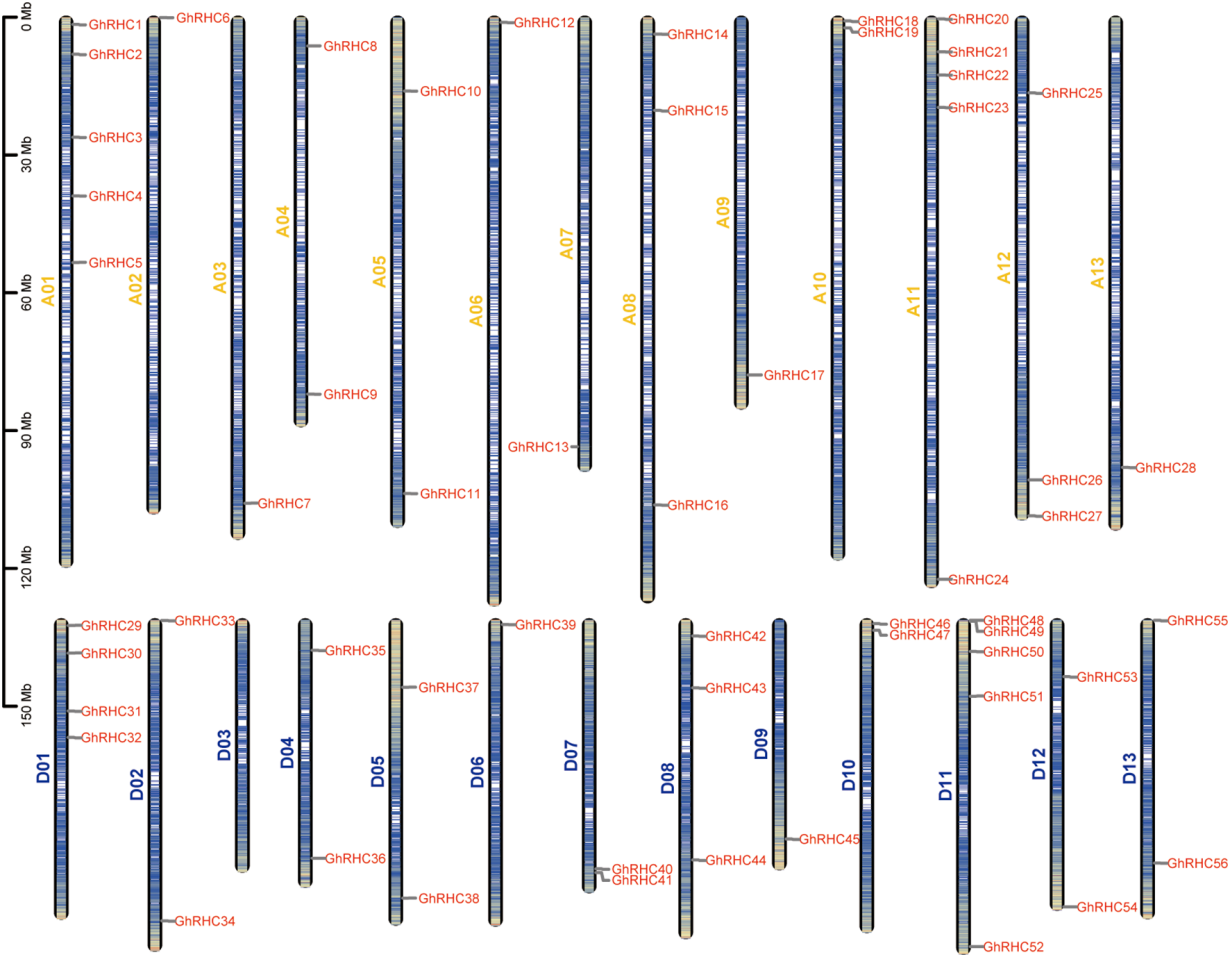
Chromosomal locations for C_3_HC_4_-type RING finger genes in cotton genomes. The length of the bar refers to the size of chromosome.

Utilizing gene annotation data, the chromosomal distribution of C_3_HC_4_-type RING finger genes was illustrated (**Figure 2**). The chromosomal localization analysis revealed that the 56 C_3_HC_4_-type RING finger genes were present on all cotton chromosomes, with the exception of chromosome D03. Notably, chromosomes A01, A11, and D11 each harbored the highest number of these genes, with five C_3_HC_4_-type RING finger genes per chromosome. Chromosome D01 contained four family members. Chromosomes A08, A12, and D08 each contained three family members. Two family members were identified on chromosomes A04, A05, A10, D02, D04, D05, D07, D10, D12, and D13. In contrast, a single family member was located on chromosomes A02, A03, A06, A07, A09, A13, D06, and D09, respectively. Among the seven sub-genomes within the A-G set of cotton, the D sub-genome is characterized by the smallest size and DNA content. Consequently, it is widely postulated that the D sub-genome represents the most primitive ancestor in the evolutionary history of the cotton genus. It is hypothesized that the other sub-genomes may have originated from the D sub-genome through an increase in DNA repeat sequences. The analogous distribution of C_3_HC_4_-type RING finger genes at corresponding loci on the A and D sub-genomes further corroborates this hypothesis. Throughout the extensive evolutionary transition from the D sub-genome to the A sub-genome, there has been minimal loss of C_3_HC_4_-type RING finger genes, underscoring their critical role in the normal growth and development of cotton.

### 3.3 Gene structure and conserved motifs of C_3_HC_4_-type RING finger genes

**Figure 3:**
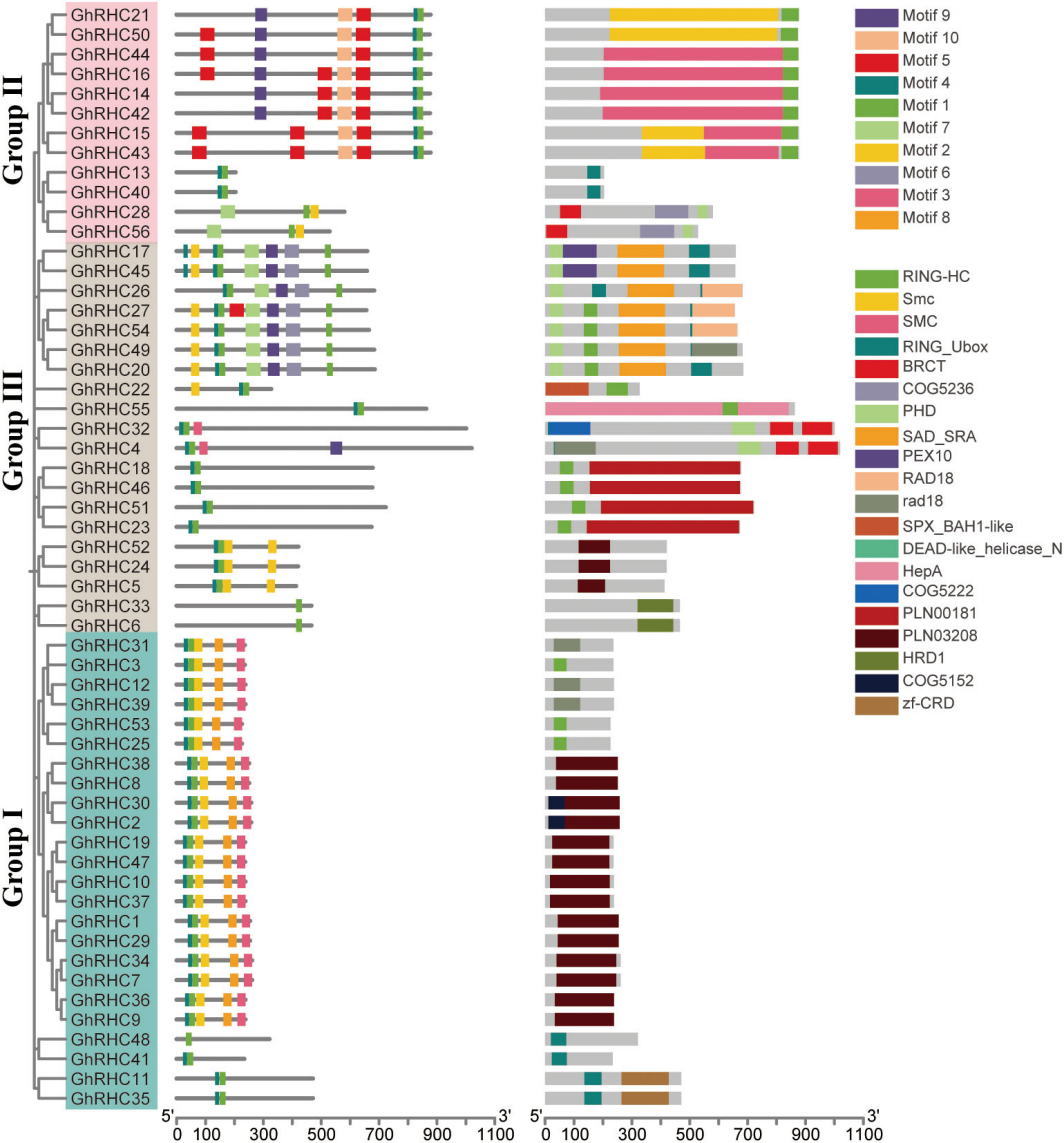
The evolutionary relationship, domain, and conserved motif analysis of C_3_HC_4_-type RING finger genes in cotton. **(A)** Evolutionary tree of C_3_HC_4_ genes. Group I, II and III were denoted by the pink, blue, and green box, respectively. **(B)** Conserved motif distribution of C_3_HC_4_-type RING finger genes. The 10 differently colored boxes represent the 10 specific motifs. **(C)** Functional domains distribution of C_3_HC_4_-type RING finger genes. The 20 differently colored boxes represent the 20 specific domains.

The conserved motifs of genes may reflect the evolutionary relationships and functional roles among members of a gene family. This study was conducted to further elucidate the characteristics of the C_3_HC_4_-type RING finger gene family by analyzing these conserved motifs. A total of ten conserved motifs were identified across 56 C_3_HC_4_-type RING finger genes, which were sequentially designated as Motif1 through Motif10 (**Figure 3**). The number of different C_3_HC_4_-type RING finger genes containing these motifs varied from one to eight. It was observed that most closely related members within Group I and Group II exhibited similar motif compositions, both in terms of number and position, suggesting that genes within these groups may share analogous functions. Motif1 is contained in all C_3_HC_4_-type RING finger genes, suggesting that Motif1 is highly conserved and has an important role in C_3_HC_4_-type RING finger genes. Motif6 is only found in Group III, Motif8 is only found in Group I, and Motif10 is only found in Group II, which is hypothesized to be a structure that may be specific to these three sections. The motif composition was both consistent and distinctive, predicting functional conservation as well as divergent roles. The domains showed the similar distribution pattern with motifs. The longer proteins contain a greater variety of domains, but all of them include the RING-HC domain. The proteins within the same cluster possessed similar C_3_HC_4_-type motifs and domains, suggesting functional similarities among the C_3_HC_4_-type proteins within the same cluster.

### 3.4 Duplication pattern of C_3_HC_4_-type RING finger genes in A and D-subgenomes

To investigate the expansion pattern, we conducted an analysis of the segmental duplication of C_3_HC_4_-type RING finger genes. Segmentally duplicated homologous blocks containing these genes were identified and are represented as gray lines in **Supplementary Figure 1**. The results indicated that these duplicated homologous blocks predominantly connected the corresponding chromosomes from the D sub-genome to the A sub-genome. In total, 70 duplicated gene pairs were identified (**Supplementary Table 5**). Among these, 24 gene pairs were located within the interior of either the A or D sub-genome, with 12 pairs in the A sub-genome and 12 pairs in the D sub-genome. Interestingly, despite both the A and B sub-genomes containing 12 pairs of duplicated genes, not all gene pairs within these specific sub-genomes exhibit direct correspondence. For instance, there is an absence of corresponding gene pairs on the D sub-genomic sequence for A01-*GhRHC3*/A06-*GhRHC12*, A03-*GhRHC7*/A04-*GhRHC9*, and A05-*GhRHC10*/A10-*GhRHC19*. Conversely, novel gene pairs have emerged on the D sub-genome, such as D01-*GhRHC29*/D02-*GhRHC34* and D02-*GhRHC34*/D04-*GhRHC36*. These findings suggest that dynamic rearrangements may have occurred subsequent to segmental duplication, resulting in the loss of certain genes.

The ratio of non-synonymous (Ka) to synonymous (Ks) mutations serves as a metric for assessing the presence of selection pressure on genes. A Ka/Ks value of 1 indicates neutral evolution, a value less than 1 suggests that the gene is subject to purifying selection, and a value greater than 1 indicates positive selection. The Ka/Ks ratios for the segmentally duplicated RHC genes ranged from 0.0444 to 0.7953, with an average of 0.2436 (refer to **Supplementary Table 6**). All Ka/Ks values for these duplications were below 1, indicating that the evolution of these genes was predominantly governed by purifying selection.

### 3.5 Expression level of C_3_HC_4_-type RING finger genes in different tissues

**Figure 4:**
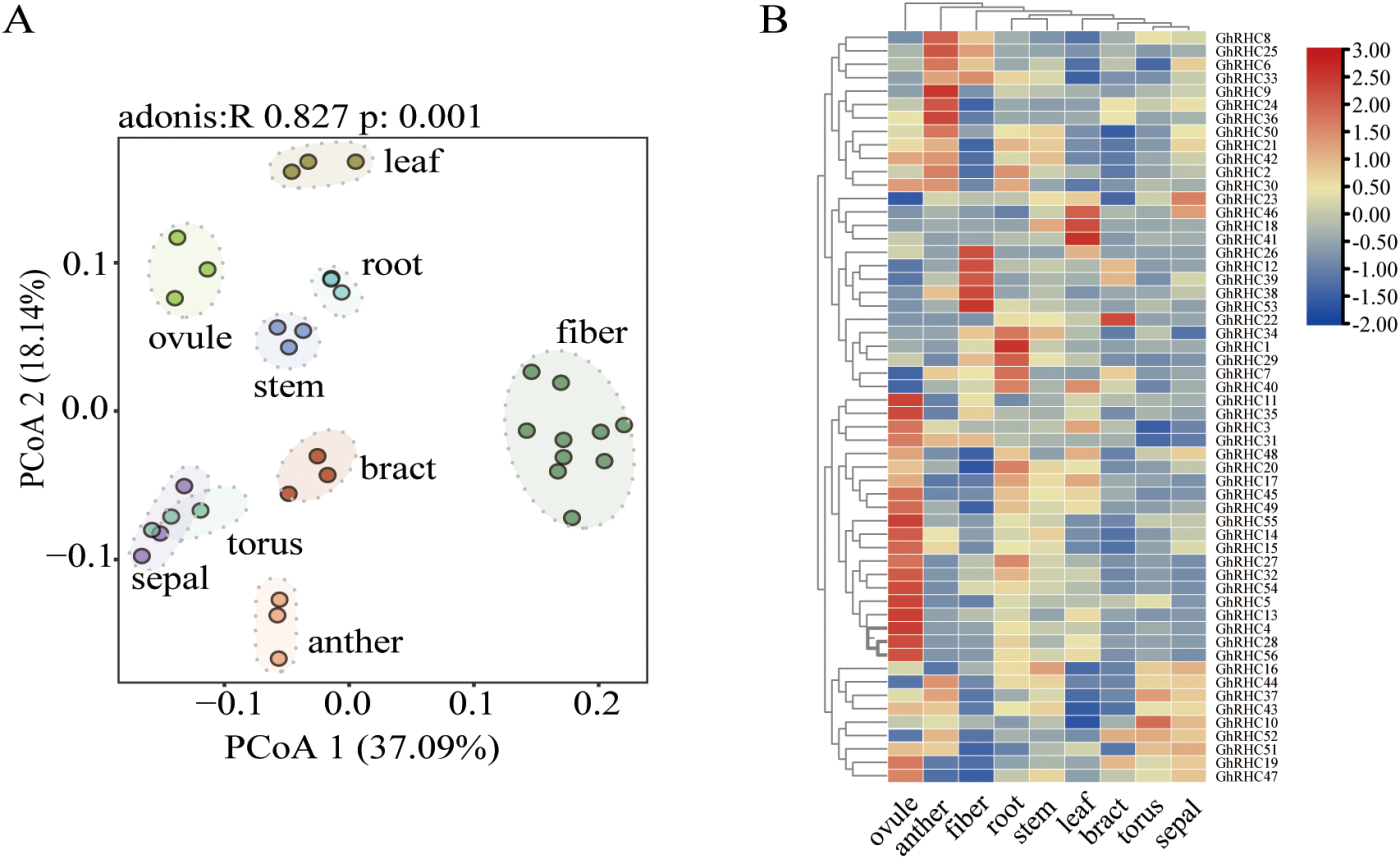
Panel **(A)** shows a PCoA plot with plant tissue samples (leaf, root,fiber, ovule, stem, bract, torus, sepal, anther) clustered by type, with statistical details (adonis R=0.827, p=0.001). Panel **(B)** displays a heatmap of gene expression for several GhRHC genes across the same tissue types, color-coded from blue (low expression) to red (high expression) with hierarchical clustering.

To explore the potential roles of C_3_HC_4_-type RING finger genes in the growth and development of cotton, their expression patterns across nine distinct tissues were analyzed using RNA-Seq data. Principal coordinates analysis (PCoA, **Figure 4A**; **Supplementary Table 7**), based on Bray–Curtis dissimilarity, effectively distinguished the samples from the nine cotton tissues. The PCoA results (R = 0.827, p-value = 0.001) accounted for 55.23% of the total variation observed. These findings suggest that C_3_HC_4_-type RING finger genes exhibit tissue-specific expression. Notably, samples from the sepal, torus, bract, and anther—components of the floral structure—formed a distinct cluster, indicating a localized expression pattern similarity among these tissues. The expression patterns illustrated by the heatmap also revealed the tissue-specific distribution of C_3_HC_4_-type RING finger genes (**Figure 4B**; **Supplementary Table 7**). Notably, four C_3_HC_4_-type RING finger genes, namely *GhRHC1*, *GhRHC7*, *GhRHC29*, and *GhRHC34*, which are categorized under Group I, exhibited high root-specific expression. Additionally, *GhRHC18*, *GhRHC41*, and *GhRHC46* demonstrated a leaf-specific expression pattern. Furthermore, over 20 C_3_HC_4_-type RING finger genes, such as *GhRHC4*, *GhRHC5*, *GhRHC13*, and *GhRHC28*, showed specific expression in ovules. This observation may be attributed to the necessity for enhanced genetic collaboration to accurately coordinate the complex developmental processes and environmental responses during the early stages of seed germination.

### 3.6 Expression of C_3_HC_4_-type RING finger genes under abiotic stress and biotic stress

**Figure 5:**
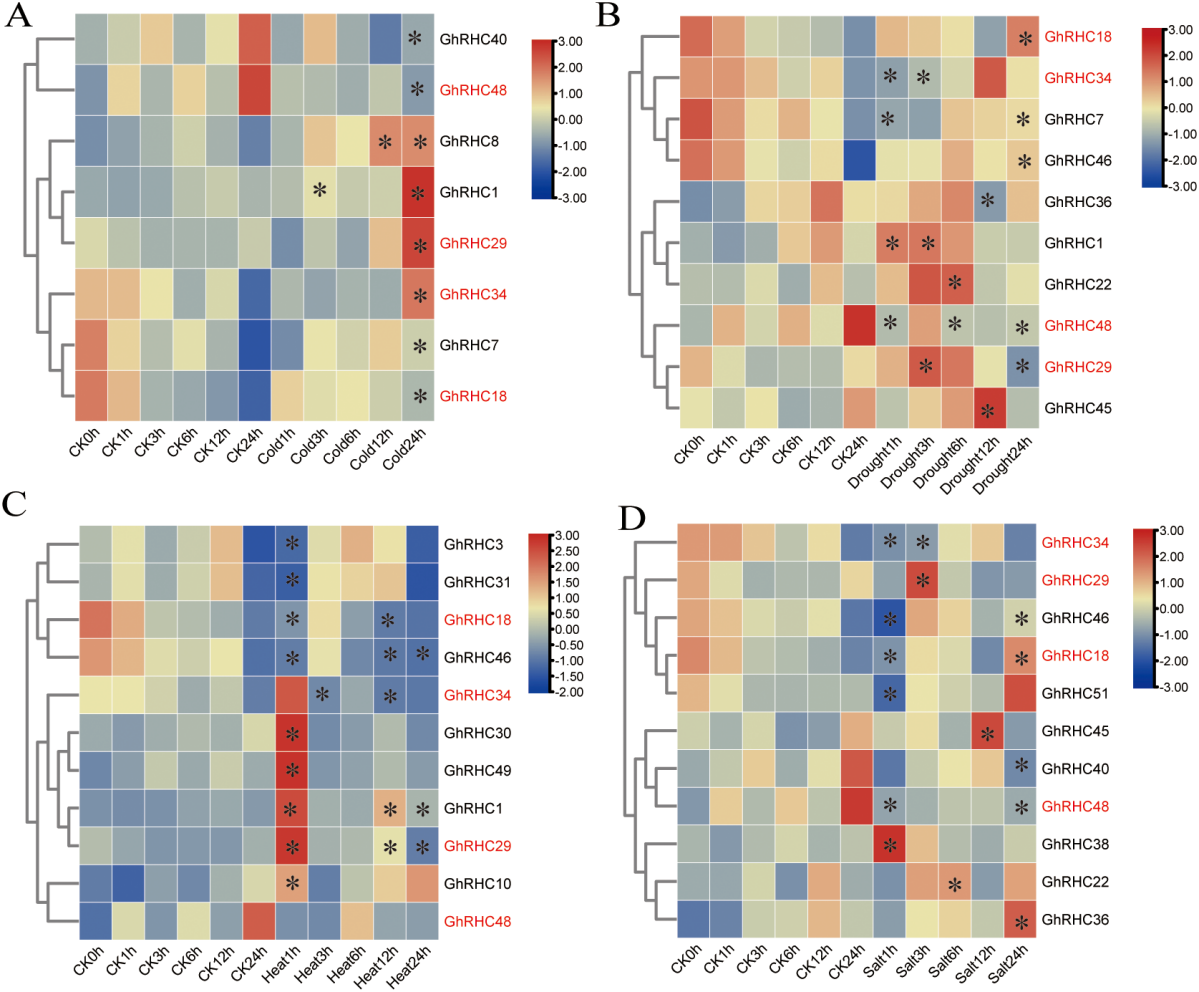
Heatmap showing the expression trends of C_3_HC_4_-type RING finger genes under cold **(A)**, drought **(B)**, heat **(C)** and salt **(D)**. * represents a significant difference (P < 0.05) compared to the CK group at the corresponding time points. Four differentially expressed C_3_HC_4_-type RING finger genes across the four stresses were marked by red font.

To explore the potential role of C_3_HC_4_-type RING finger genes in response to abiotic stress, the expression patterns of these genes in cotton were examined under cold, drought, heat, and salt conditions. Principal Coordinates Analysis (PCoA) based on Bray–Curtis dissimilarity indicated that cold treatment (**Supplementary Figure 2A**) significantly altered the expression patterns of RHC members, more so than drought (**Supplementary Figure 2B**), heat (**Supplementary Figure 2C**), or salt (**Supplementary Figure 2D**) treatments. The small R values suggest that RHC members exhibit insensitivity to abiotic stressors. A heatmap analysis revealed that 8, 10, 11, and 11 C_3_HC_4_-type RING finger genes exhibited significant expression changes under cold, drought, heat, and salt treatments, respectively (**Figure 5**; **Supplementary Tables 8**–**S11**). Notably, the expression levels of *GhRHC18*, *GhRHC29*, *GhRHC34*, and *GhRHC48* were significantly altered at least at one time point across the four types of abiotic stress. It is noteworthy that the expression level of *GhRHC1* was significantly affected by cold, drought, and heat stress.

**Figure 6:**
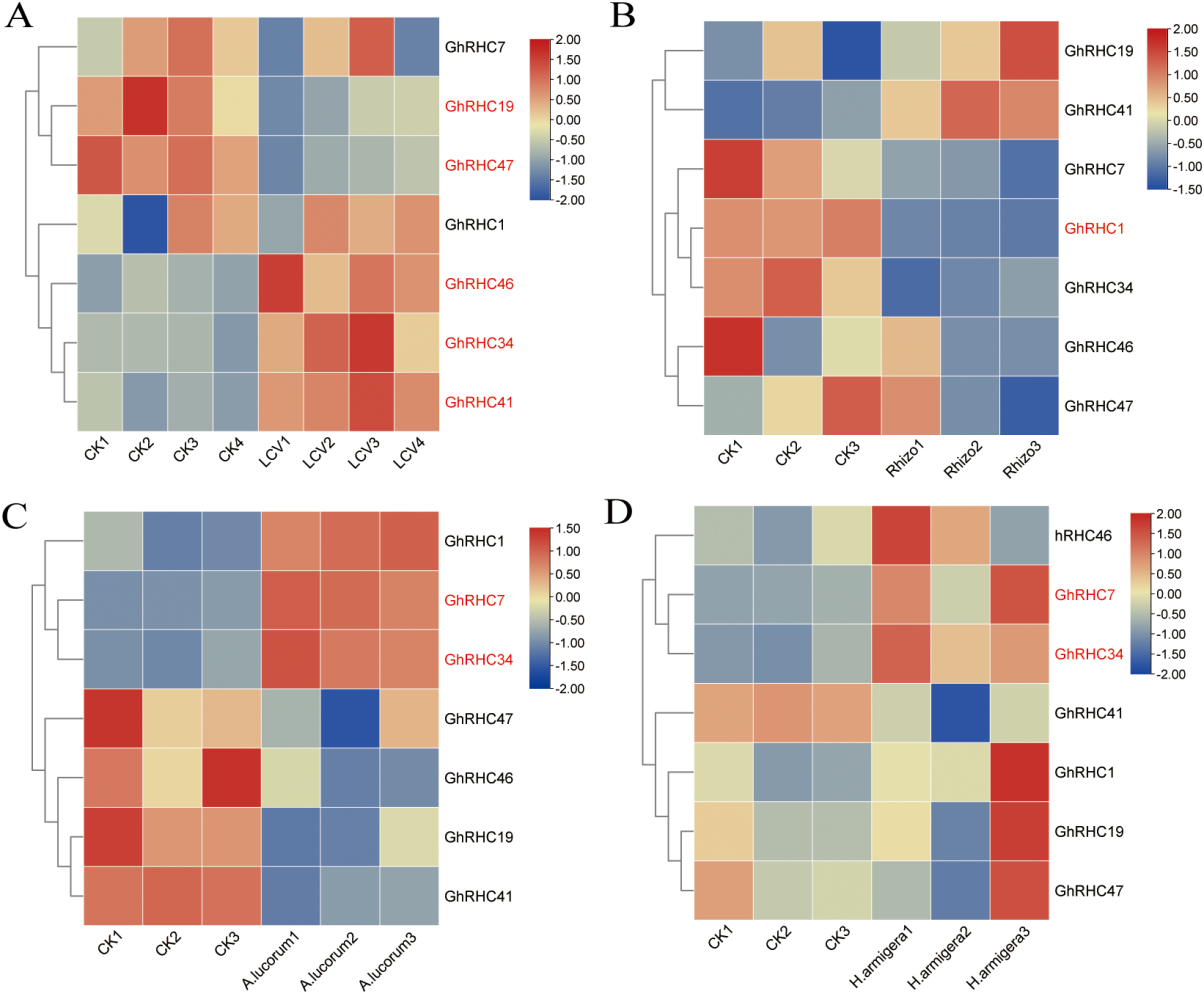
Heatmap showing the expression trends of C_3_HC_4_-type RING finger genes under leaf curl virus **(A)**, *Rhizobacteria* **(B)**, *A. lucorum* **(C)** and *H. armigera* **(D)** treatments. Differentially expressed C_3_HC_4_-type RING finger genes across the four stresses were marked by red font.

To elucidate the potential role of C_3_HC_4_-type RING finger genes in response to biotic stress, we analyzed the expression patterns of these genes in cotton subjected to various biotic stressors, including leaf curl virus (**Supplementary Figure 3A**), Fusarium wilt (**Supplementary Figure 3B**), Rhizobacteria (**Supplementary Figure 3C**), and the insect pests Apolygus lucorum and Helicoverpa armigera (**Supplementary Figure 3D**). Principal Coordinates Analysis (PCoA) results indicated that both leaf curl virus and pest infestations significantly affected the expression of C_3_HC_4_-type RING finger genes. In total, seven C_3_HC_4_-type RING finger genes—namely, *GhRHC1*, *GhRHC7*, *GhRHC19*, *GhRHC34*, *GhRHC41*, *GhRHC46*, and *GhRHC47*—exhibited significant expression changes under biotic stress conditions. Specifically, leaf curl virus upregulated the expression of *GhRHC1*, *GhRHC34*, *GhRHC41*, and *GhRHC36*, while downregulating *GhRHC19* and *GhRHC47* (**Figure 6A**; **Supplementary Tables 12**–**S15**). Additionally, the insect pests *A. lucorum* and *H. armigera* consistently upregulated the expression of *GhRHC7* and *GhRHC34* (**Figures 6C, D**). Interestingly, the genes *GhRHC1*, *GhRHC7*, and *GhRHC34* exhibited upregulated expression in response to biotic stress. Conversely, these genes were downregulated in the presence of Rhizobacteria, as illustrated in **Figure 6B**. These findings suggest that *GhRHC1*, *GhRHC7*, and *GhRHC34* may play significant roles in mitigating the detrimental effects of biotic stress. A comprehensive analysis of the expression levels of C_3_HC_4_-type RING finger genes indicates that *GhRHC1* and *GhRHC34* are responsive to both biotic and abiotic stressors. While these transcriptomic findings are robust and consistent with the known roles of RING finger proteins as general stress modulators, focused qRT-PCR validation was conducted on cold stress to confirm the technical reliability of the expression data.

### 3.7 Relative expression level and subcellular localization of *GhRHC1* and *GhRHC34*

**Figure 7:**
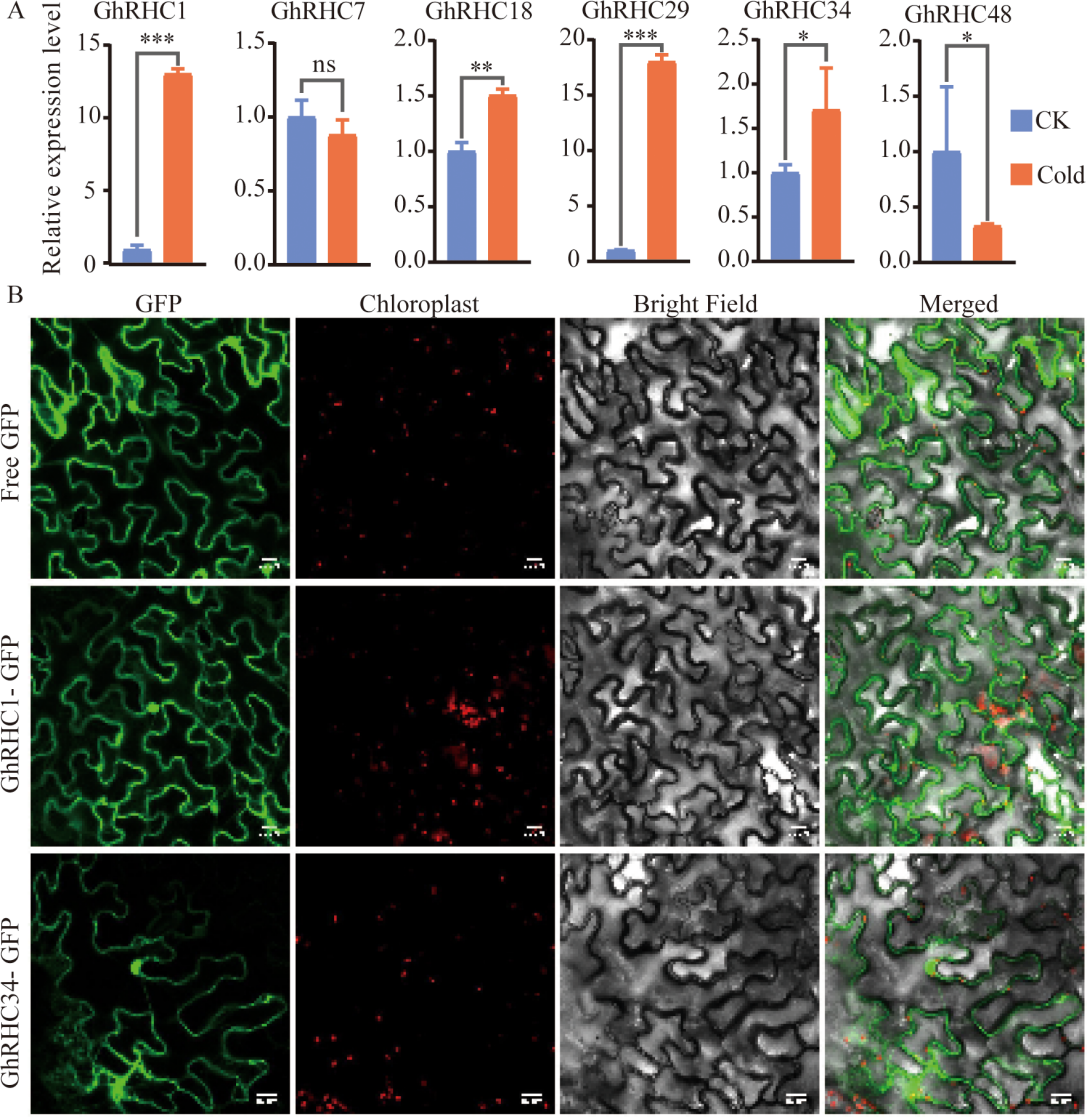
Relative expression levels of C_3_HC_4_-type RING finger genes determined by qRT-PCR in cotton leaves under cold treatment **(A)** and subcellular localization of GhRHC1 and GhRHC34 proteins in *Nicotiana benthamiana* leaves **(B)**. In part A, significant differences were determined by Wilcoxon rank sum test (* *P* < 0.05; ** *P* < 0.01; *** *P* < 0.001, ns *P* > 0.05). In part B, each protein was fused with GFP and driven by the 35S promoter. The localization of chloroplasts is marked by the self- luminescence of chloroplasts. Scale bars = 20 μm.

To validate the accuracy of transcriptome data, six differently expressed *GhRHC* genes (*GhRHC1/7/18/29/34/48*) were selected for qRT-PCR. Results showed that the expression levels of *GhRHC1*, *GhRHC18*, *GhRHC29* and *GhRHC34* were significantly promoted after a 24-hour cold treatment (**Figure 7A**). These results were consistent with the transcriptome data. To investigate the subcellular localization, full-length CDSs of *GhRHC1* and *GhRHC34* without termination codons were cloned into the pCAMBIA1302 vector with an enhanced green fluorescent protein (GFP) tag under the 35 S promoter via LR reaction (**Figure 7B**). The empty GFP vectors was set as the control group. Three days after infiltrating into the leaf epidermal cells of tobacco, localization of *GhRHC1* and *GhRHC34* were observed by the confocal microscope. The results show that the two proteins, *GhRHC1* and *GhRHC34*, can be preliminarily identified as being distributed in both the nucleus and the cytoplasm. Specific nuclear and cytoplasmic markers are indispensable to obtain the subcellular localization of *GhRHC1* and *GhRHC34* in the future work.

## 4 Discussion

### 4.1 Evolutionary characteristics and structural diversity of the *GhRHC* gene family

As pivotal components of E3 ubiquitin ligases, C_3_HC_4_-type RING finger proteins are ubiquitous in eukaryotes and play indispensable roles in regulating plant growth, development, and stress adaptation (Su et al., 2024). In this study, 56 *GhRHC* genes were identified in the *Gossypium hirsutum* genome. This number is comparable to the family sizes reported in rice (Lim et al., 2010) and *Arabidopsis* (Kosarev et al., 2002), suggesting that the *GhRHC* family has maintained a relatively stable scale throughout plant evolution. Phylogenetic analysis categorized these members into three distinct subfamilies (Groups I, II, and III). While members within each subfamily exhibited high consistency in gene structure and protein length, significant divergence was observed between subfamilies. For instance, Group I members possessed the fewest introns and the shortest average protein length, whereas Groups II and III exhibited more complex structures. This structural divergence suggests that the *GhRHC* gene family has undergone functional diversification during evolution to meet complex biological demands (Lemke et al., 2025).

Motif analysis further corroborated the reliability of this classification. Motif1 was present in all members, indicating it is a core component of the C_3_HC_4_ domain essential for maintaining protein function. Conversely, Groups I, II, and III contained specific motifs (Motif8, Motif10, and Motif6, respectively). The retention of these subfamily-specific motifs may confer distinct substrate recognition capabilities or regulatory mechanisms (Pereira-Leal and Seabra, 2000). Furthermore, chromosomal localization and synteny analysis revealed an enrichment of *GhRHC* genes at chromosomal ends and extensive homologous correspondence between the D-subgenome and the A-subgenome. Ka/Ks analysis indicated that all duplicated gene pairs were subject to purifying selection (Ka/Ks < 1). This demonstrates that despite polyploidization events in upland cotton, the functions of the *GhRHC* gene family have remained highly conserved under evolutionary pressure, with deleterious mutations being eliminated to maintain their fundamental roles in cotton growth and development (Cardoso et al., 2016).

### 4.2 *GhRHC* genes exhibit significant tissue specificity, particularly in root and ovule development

Spatiotemporal expression patterns are intrinsically linked to biological function. This study revealed distinct tissue-specific expression profiles for *GhRHC* genes. Notably, Group I members *GhRHC1*, *GhRHC7*, *GhRHC29*, and *GhRHC34* exhibited highly root-specific expression. As roots are the primary organ for nutrient and water absorption and for sensing the soil environment, the high expression of these genes implies their potential involvement in establishing root system architecture or mediating signal transduction in the rhizosphere (An et al., 2012). Additionally, over 20 *GhRHC* members (e.g., *GhRHC4*, *GhRHC13*) were specifically enriched in ovules. Cotton fibers are single-celled trichomes differentiated from ovule epidermal cells; thus, ovule development directly determines fiber yield and quality (Yuan et al., 2021). Previous studies have established that the ubiquitin pathway participates in the regulation of plant reproductive organ development (Moon et al., 2004). The enrichment of numerous *GhRHC* genes in ovules strongly suggests that these members may precisely regulate early ovule development and fiber cell initiation by mediating the degradation of specific substrates, providing potential candidate genes for the genetic improvement of cotton fiber traits.

### 4.3 Pleiotropy of *GhRHC* genes in response to abiotic stress

Abiotic stress is a major limiting factor for cotton yield (Hassan et al., 2020). As E3 ubiquitin ligases, RING finger proteins often degrade negative regulators via the ubiquitin-proteasome system (UPS) to activate stress signaling pathways (Qu et al., 2021). Our results indicate that cold stress had the most significant impact on *GhRHC* gene expression, followed by drought, heat, and salt stress. This aligns with findings in other species, reinforcing the core role of C_3_HC_4_-type zinc finger proteins in cold adaptation (Kim and Kang, 2006; Han et al., 2021). Of particular interest is the observation that *GhRHC18*, *GhRHC29*, *GhRHC34*, and *GhRHC48* responded to multiple abiotic stresses, exhibiting significant pleiotropy. This broad-spectrum response capability suggests that these genes may act as nodes of crosstalk between different stress signaling pathways, making them ideal targets for genetic engineering to enhance stress resistance. For example, *GhRHC1* showed significant expression changes under cold, drought, and heat stress, suggesting it may function as a general stress response regulator assisting cotton in maintaining cellular homeostasis.

### 4.4 The “Double-Edged” role of *GhRHC* genes in pathogen defense and beneficial symbiosis

Plants must deploy distinct immune programs when facing pathogen invasion versus beneficial microbial symbiosis (Delaux and Schornack, 2021). A significant finding of this study is the unique expression pattern of *GhRHC* genes under biotic stress. *GhRHC1*, *GhRHC7*, and *GhRHC34* were significantly upregulated upon exposure to Leaf curl virus and insect pests (*Helicoverpa armigera*, *Apolygus lucorum*), indicating they are transcriptionally responsive to biotic stress conditions (Cheng et al., 2025). However, an intriguing phenomenon was observed: these same genes (*GhRHC1*, *GhRHC7*, *GhRHC34*) were downregulated in the presence of beneficial *Rhizobacteria*. This contrasting expression trend reveals a sophisticated “trade-off” mechanism within the plant immune system: to permit the colonization and symbiosis of beneficial microbes, the plant must actively suppress specific immune responses (Karasov et al., 2017). If the primary function of genes like *GhRHC1* is to activate immunity, their downregulation during the initial stages of symbiosis would favor bacterial survival. This suggests that *GhRHC1*, *GhRHC7*, and *GhRHC34* are not only key genes for disease and insect resistance but also critical switches regulating the plant-microbe interaction balance. Combined with their high expression in roots, we hypothesize that they function as critical “gatekeepers” at the root-microbe interface.

### 4.5 Subcellular localization of GhRHC1 and GhRHC34 provides functional support for their regulatory roles

Subcellular localization analysis showed that GhRHC1 and GhRHC34 are distributed in both the nucleus and cytoplasm. This localization pattern is consistent with the canonical functions of C_3_HC_4_-type RING finger proteins as E3 ubiquitin ligases, which often regulate substrates in multiple cellular compartments (Barroso-Gomila et al., 2023). Nuclear localization suggests potential involvement in the ubiquitination of transcriptional regulators or other nuclear proteins associated with stress-responsive gene expression, whereas cytoplasmic localization implies roles in post-translational regulation of signaling components during stress perception and signal transduction (Goswami et al., 2024; Hernández-Elvira and Sunnerhagen, 2022). Importantly, the nucleus–cytoplasm distribution of GhRHC1 and GhRHC34 is in agreement with their broad transcriptional responsiveness to both abiotic and biotic stresses. Such dual localization may facilitate coordinated regulation between upstream stress signaling in the cytoplasm and downstream transcriptional control in the nucleus (Bilen et al., 2022). Combined with their root-enriched expression and contrasting regulation during pathogen attack versus rhizobacterial colonization, these results support the view that GhRHC1 and GhRHC34 function as versatile regulatory nodes involved in balancing stress defense and plant–microbe interactions in cotton. It should be noted that subcellular localization was examined under non-stress conditions, and whether GhRHC1 and GhRHC34 undergo stress-induced re-localization requires further investigation.

## 5 Conclusion

In summary, we performed a comprehensive genome-wide identification and analysis of the C_3_HC_4_-type RING finger gene family in *Gossypium hirsutum*. The family is evolutionarily conserved but exhibits significant divergence in structure and expression patterns. Expression profiling identified key candidate genes involved in ovule development and highlighted the critical role of *GhRHC* members in abiotic stress responses, particularly cold stress. Most importantly, we discovered that core members such as *GhRHC1* and *GhRHC34* not only respond to multiple abiotic stresses but also display antagonistic regulatory patterns between pathogen defense and beneficial symbiosis. These findings enrich our understanding of C_3_HC_4_-type zinc finger protein functions in cotton and provide candidate genes for future functional validation and molecular breeding studies. that are high-yielding, broadly stress-resistant, and capable of efficiently utilizing rhizosphere microorganisms.

## Data Availability

The datasets presented in this study can be found in online repositories. The names of the repository/repositories and accession number(s) can be found in the article/**Supplementary Material**.

## Supplementary material

Supplementary Figure 1 Duplication pattern of the RHC members. The red lines indicate the homology of *GhRHC* genes. Segmental duplicated homologous blocks are indicated by gray lines. Chromosome numbers are displayed at the bottom of each chromosome.

Supplementary Figure 2 Principal coordinates analysis (PCoA) of C_3_HC_4_-type RING finger genes under cold **(A)**, drought **(B)**, heat **(C)** and salt **(D)** based on Bray–Curtis distances Significant differences among the plant compartments were determined using PERMANOVA.

Supplementary Figure 3 Principal coordinates analysis (PCoA) of C_3_HC_4_-type RING finger genes under leave curl virus **(A)**, *Fusarium* wilt **(B)**, *Rhizobacteria* **(C)**, *Apolygus lucorum* and *Helicoverpa armigera* **(D)** treatments based on Bray–Curtis distances Significant differences among the plant compartments were determined using PERMANOVA.
