# Supplementary figures and images for: Genome-wide identification and expression analysis of C3HC4-type RING finger gene family in Gossypium hirsutum
Source: Front Plant Sci. 2026 May 12;17:1789503. doi: 10.3389/fpls.2026.1789503 (PMC13201221; doi:10.3389/fpls.2026.1789503)

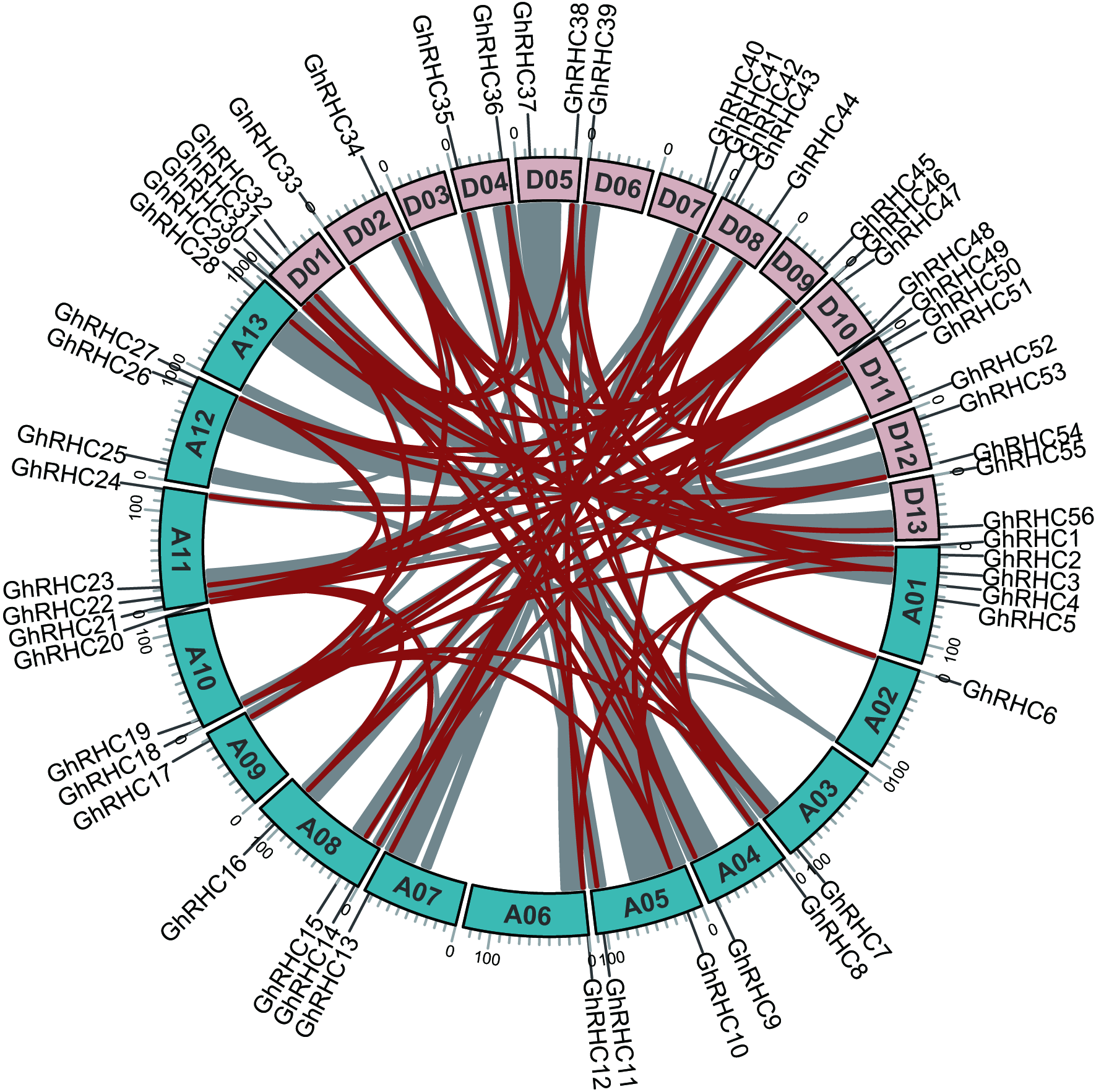

Supplement: Supplementary file 1 [file Image1.tif]

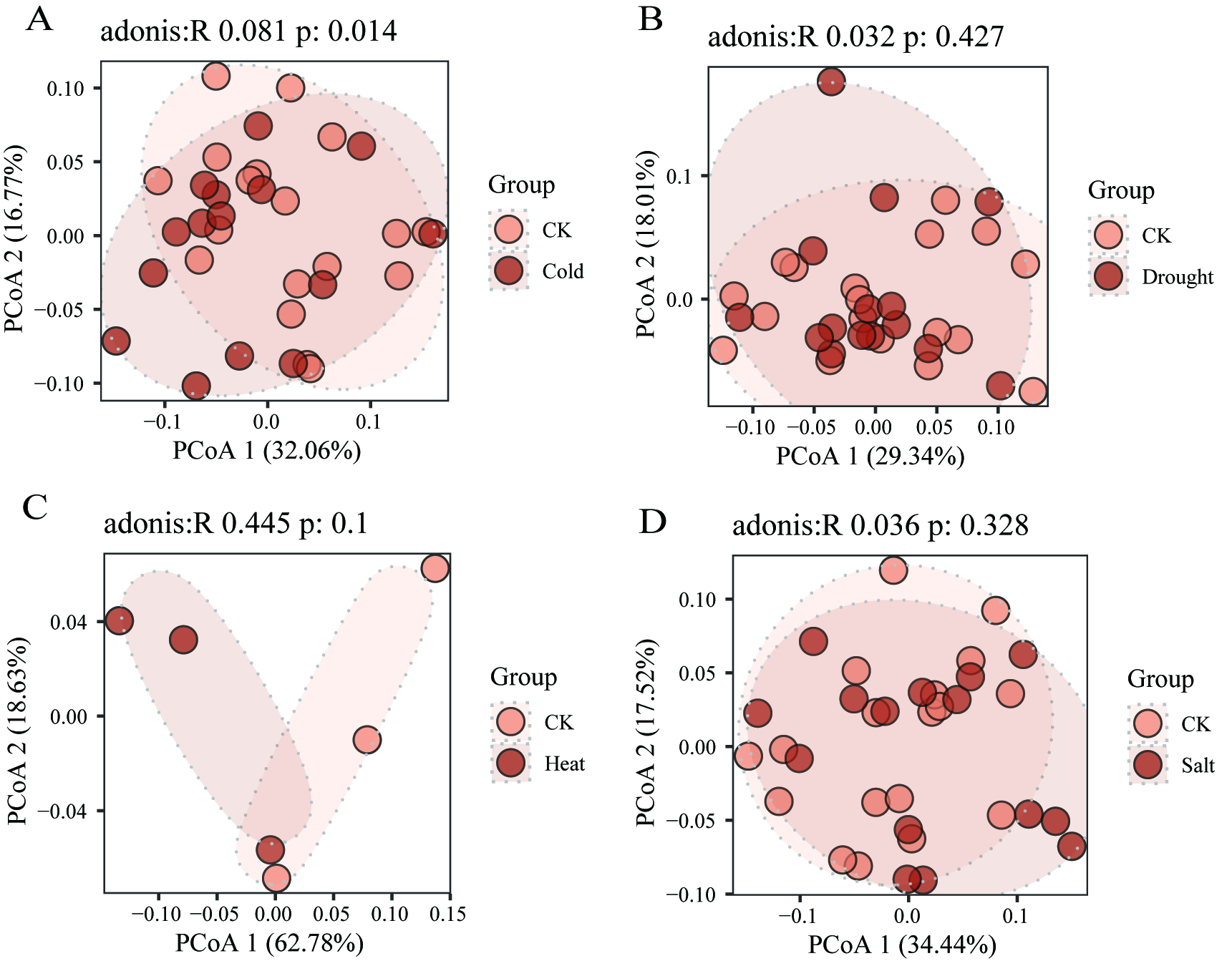

Supplement: Supplementary file 2 [file Image2.tif]

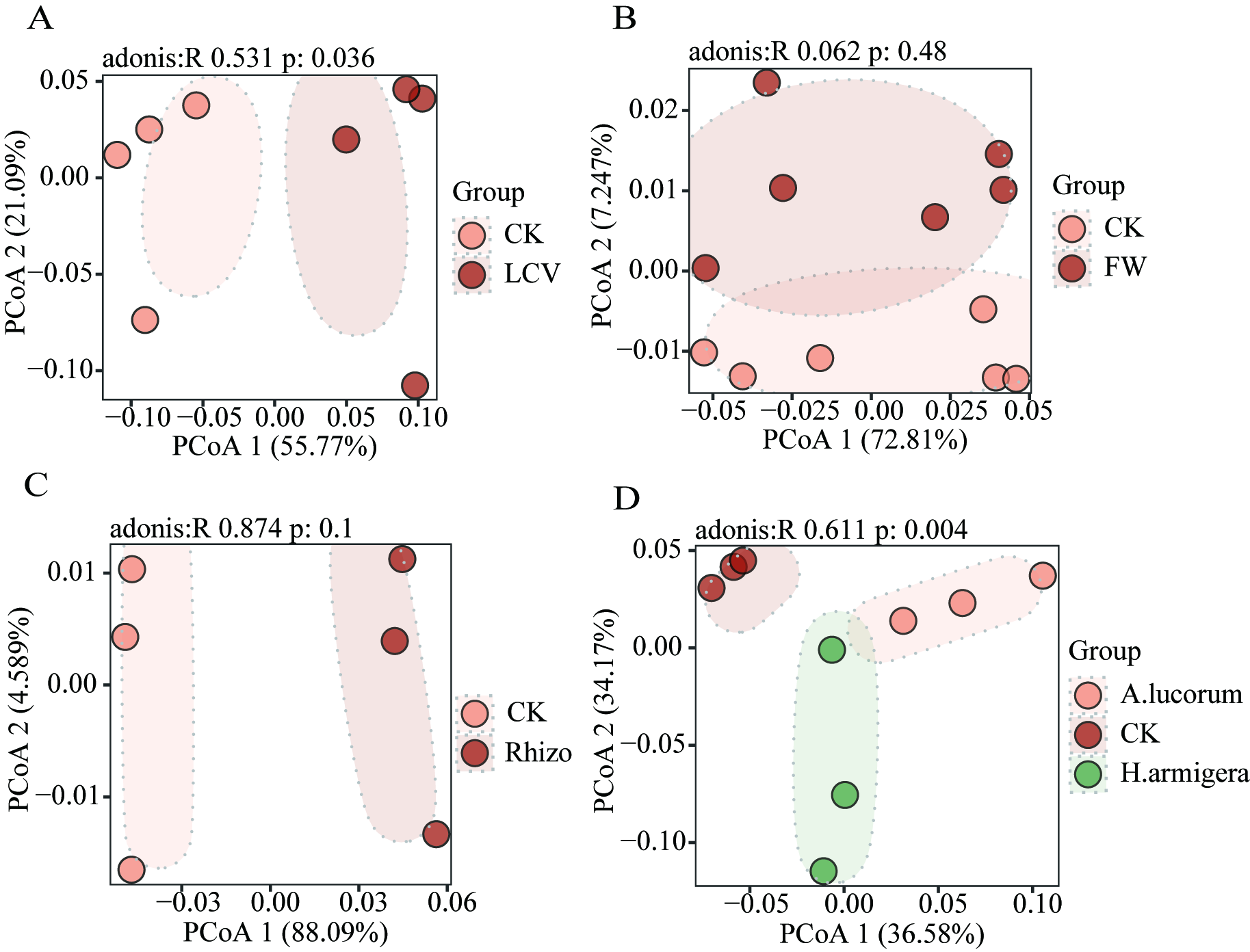

Supplement: Supplementary file 3 [file Image3.tif]
